# Minutes of the Ohio State Dental Society

**Published:** 1868-06

**Authors:** G. W. Keely

**Affiliations:** Columbus, Ohio


### Proceedings of Societies.

---

### MINUTES OF THE OHIO STATE DENTAL SOCIETY.

COLUMBUS, Ohio, May 20, 1868.

SOCIETY met pursuant to a call by the President, at 10½ o'clock A. M., for the purpose of consultation, and the adoption of such measures as may be deemed best, for the execution of the law recently enacted by the Legislature of the State, "To regulate the Practice of Dentistry." Dr. Taft, President in the chair. G. W. Keely was appointed Secretary, *pro tem.*

On calling the roll of members the following named were present: G. W. Keely, J. Taft, H. A. Smith, A. Berry, N. W. Williams, A. A. Blount, M. DeCamp, J. McKinley, J. Fowler, W. M. Herriott, J. C. Whinnery, G. W. Dunn, A. R. Lord, I. Williams, W. E. Dunn, F. Emons, H. M. Edson.

On motion, those of the profession present, not members, were invited to participate in the discussion. Dr. H. A. Smith offered the following resolution, which was odopted: "That a committee of three be appointed, (the President being Chairman) to prepare a circular to be addressed to a committee of one in each county in the State, for the purpose of obtaining a correct list of Dentists in practice, and their students; said list to become a part of the records of tl e society. H. A. Smith and M. DeCamp, were appointed to fill the committee. The following report was offered by the committee, and adopted:

DEAR SIR: At a meeting of the Ohio State Dental Society, held at Columbus, on the 20th ult., the undersigned were appointed a committee to obtain the names of all practitioners and students of Dentistry in the State.

The object in procuring these names is to aid the State Society to carry out the provisions of the law recently enacted to "Regulate the Practice of Dentistry in the State of Ohio," a copy of which is herein inclosed.

To aid this object, will you please fill the following blank, and inclose the same to the Chairman of the Committee at as early a day as convenient.

Names of Practitioners of Dentistry in ——— county.

How long in practice ——. Where located ——.

Names of Students of Dentistry ———.

Respectfully,

J. Taft,<br>

M. DeCamp, } Committee.<br>

H. A. Smith,

Dr. A. A. Blount, offered the following resolution, "That a Committee of five be appointed to select proper persons who shall constitute the Board of Examiners, whose duty it shall be to examine all applicants for certificates for the practice of Dentistry, under the law regulating the same. Drs. Blount, DeCamp, Berry, Whinnery and Herriott were selected as Committee.

On motion, it was resolved that the Examining Board consist of five members, three of whom shall constitute a quorum.

Society adjourned to meet at 1½ P. M.

1½ P. M.—Society met; minutes of morning session read and approved.

On motion of Dr. DeCamp, Drs. Keely, Berry, and H. A. Smith were appointed to procure a Seal for the Society, and Certificates to be conferred by the Examining Board.

It was resolved that the sum of twenty-five dollars be charged for certificates, which amount must be paid into the treasury on or before the delivery of the same.

*Report of Committee to select members of Board of Examiners, with suggestions, which was adopted:*

Your Committee, appointed to select a Board of Examiners, would respectfully report the following names:

For 3 years, Dr. J. Taft, of Cincinnati.
" " Dr. F. H. Rehwinkle, of Chillicothe.
" 2 " Dr. M. DeCamp, of Mansfield.
" " Dr. W. P. Horton, of Cleveland.
" 1 " Dr. C. H. Harroun, of Toledo.

Respectfully submitted,

A. A. Blount,

W. M. Herriott,

A. Berry,

M. DeCamp,

J. C. Whinnery, } Committee.

Your Committee would suggest that two members of this Committee should serve for the term of three years, two for two years, and one for one year. In the event of the death or resignation of any member of this Board of Examiners, the remaining members, together with the officers of the State Society be empowered to fill such vacancy, until a regular meeting of the Society.

A lively and interesting discussion sprung up in regard to necessary qualifications of those to whom certificates should be given, participated in by Drs. Herriott, Berry, Smith, Whinnery, DeCamp, Dunn, Keely, Williams, Blount, and others.

On motion, the Board of Examiners were allowed ten dollars per day and expenses, during the time engaged in such duty.

It was unanimously resolved, That the thanks of this Society be tendered to Hon. G. W. Brooke, and others, who were instrumental in procuring the passage of the law "To Regulate the Practice of Dentistry," after which the Society adjourned.

G. W. Keely, *Secretary.*

